# Evidence of STLV 2 and STLV 3 infections in wild living bonobos (P. paniscus ) from the Democratic Republic of Congo

**DOI:** 10.1186/1742-4690-8-S1-A93

**Published:** 2011-06-06

**Authors:** Steve Ahuka-Mundeke, Florian Liegeois, Octavie Lunguya, Valentin Mbenzo, Menard Mbende, Bila-Isia Inogwabini, Ahidjo Ayouba, Jean-Jacques Muyembe, Eric Delaporte, Martine Peeters

**Affiliations:** 1 UMI 233, Institut de Recherche pour le Developpement (IRD) and University of Montpellier 1, Montpellier, France; 2 National Institute of Biomedical Research , Kinshasa, Democratic Republic of Congo; 3 Lac Tumba Project, WWF, Kinshasa,Democratic Republic of Congo

## Background

Among the four types of HTLV (1,to 4), only three have their simian counterparts (STLV-1, 2 and 3). STLV-1 and 3 have been found in a large number of captive and wild-living monkeys and great apes from Africa and Asia. STLV-2 was reported only in a limited number of captive bonobos (P. paniscus) from Democratic Republic of Congo (DRC), but so far never documented in wild-living populations.

## Material and methods

Between March and July 2010, fecal samples from wild-living bonobos were collected at Malebo forest in the Bandundu province, western DRC. We confirmed the species by mitochondrial (mt) DNA analyses and we screened all samples for STLV infection using a generic PCR allowing amplification of partial fragment in tax (220bp) as well as targeting different fragments in the LTR.

## Results

We collected a total of 268 bonobos fecal samples. Overall, 3 (1.1%) samples amplified the tax fragment. Among these, one (Pp5538) was identified as STLV-2 and two (Pp5489 and Pp5560) as STLV-3 by phylogenetic analysis. Additional analysis on the LTR fragment showed that the new STLV-2 from Pp5538 clustered with STLV-2 strains previously described in captive bonobos. All PCR attempts to amplify the LTR fragment of Pp5489 and 5560 samples were unsuccessfull.

## Conclusion

Our study shows that faecal samples can be used to screen for STLV infection in endangered apes, although most likely with lower sensitivities. We confirmed STLV-2 infection in wild-living bonobos and showed that bonobos are also infected with STLV-3.

